# Machine Learning-Based Crop Stress Detection in Greenhouses

**DOI:** 10.3390/plants12010052

**Published:** 2022-12-22

**Authors:** Angeliki Elvanidi, Nikolaos Katsoulas

**Affiliations:** Laboratory of Agricultural Constructions and Environmental Control, Department of Agriculture Crop Production and Rural Environment, University of Thessaly, Fytokou Str., 38446 Volos, Greece

**Keywords:** photosynthesis rate, soft sensor, gradient boosting, multilayer perceptron, microclimate

## Abstract

Greenhouse climate control systems are usually based on greenhouse microclimate settings to exert any control. However, to save energy, water and nutrients, additional parameters related to crop performance and physiology will have to be considered. In addition, detecting crop stress before it is clearly visible by naked eye is an advantage that could aid in microclimate control. In this study, a Machine Learning (ML) model which takes into account microclimate and crop physiological data to detect different types of crop stress was developed and tested. For this purpose, a multi-sensor platform was used to record tomato plant physiological characteristics under different fertigation and air temperature conditions. The innovation of the current model lies in the integration of photosynthesis rate (Ps) values estimated by means of remote sensing using a photochemical reflectance index (PRI). Through this process, the time-series Ps data were combined with crop leaf temperature and microclimate data by means of the ML model. Two different algorithms were evaluated: Gradient Boosting (GB) and MultiLayer perceptron (MLP). Two runs with different structures took place for each algorithm. In RUN 1, there were more feature inputs than the outputs to build a model with high predictive accuracy. However, in order to simplify the process and develop a user-friendly approach, a second, different run was carried out. Thus, in RUN 2, the inputs were fewer than the outputs, and that is why the performance of the model in this case was lower than in the case of RUN 1. Particularly, MLP showed 91% and 83% accuracy in the training sample, and 89% and 82% in testing sample, for RUNs 1 and 2, respectively. GB showed 100% accuracy in the training sample for both runs, and 91% and 83% in testing sample in RUN 1 and RUN 2, respectively. To improve the accuracy of RUN 2, a larger database is required. Both models, however, could easily be incorporated into existing greenhouse climate monitoring and control systems, replacing human experience in detecting greenhouse crop stress conditions.

## 1. Introduction

The predictions for further population growth up to 20% by 2050 lead to increased requirements for food production. That is why the greatest challenge for a sustainable future in the food sector is to produce more products per hectare without further expansion of the agricultural land and with less environmental impact [1]. In this regard, there is a need for more circular cropping systems, such as greenhouses and especially hydroponics.

In hydroponics, where plants are cultivated in soilless systems and in a closed environment, water and nutrients are recycled with a high degree of circularity [2,3]. However, the existing hydroponic systems are not capable of feeding the growing population. The majority of greenhouse crops are grown in the soil, and to improve productivity, it may be needed to convert more conventional greenhouses into high-tech and sophisticated systems, while the climate and fertigation control systems of the existing low-tech greenhouses should be redesigned and adopted to increase the sustainability of these production systems.

To date, the greenhouse climate and fertigation control is performed on the basis of a wide variety of environmental controllers supported by automated control models. Recently, Machine Learning (ML) algorithms have been added in these models to make controllers more efficient. Adjusting ML methodologies further reduced agrochemicals, energy and water inputs to the greenhouse system [3].

However, most of the control systems involve only climate data and do not take into account the actual physiological status of the crop. The reason of this is that, so far, crop physiological parameters were measured using time-consuming protocols that are difficult to apply remotely and in real time. The only physiological parameter adjusted up to now in recent ML models is leaf temperature [4,5]. It is the only factor that could be measured remotely providing a database large enough to build a ML model. However, leaf or crop temperature is an unstable factor which may show an intense variation through the day. As a result, it cannot be used on its own to estimate different types of crop stress [6]. There is a need to add an extra crop physiological parameter that could aim to clarify the crop physiological status. The combination of leaf temperature and photosynthesis rate (Ps) variation over the day could improve the methodology of detecting a physiological stress present in the crop.

Recently, a series of agricultural-applied ML models were developed using digital or hyperspectral data to estimate crop physiological parameters [7,8]. For instance, Ferentinos [9] developed a standard deep learning model for plant disease identification through visual observations of plant foliage. The model was based on specific convolutional neural network architectures.

The majority of these models [10,11,12], however, are applicable in open-field crops by collecting spatial–spectral data by images recorded either from satellites and drones or from optical sensors mounted on agricultural tractors. However, this is not the case for greenhouse crops; but they cannot be used for real-time stress detection, as complex protocols should be adjusted to the process. In general, the greenhouse structure and the complex lighting and shading conditions around the crop surface affect the collected image data. Consequently, the development of climate and crop physiological status monitoring and control systems for greenhouses is still challenging.

Recently, ‘soft’ sensors (i.e., mathematical models using real-time sensor data) capable of measuring various crop physiological parameters with the potential to create a time-series database have been developed [13]. The key is to adjust the spectral–spatial information obtained by the hyperspectral images into soft sensors protocol. With the recent integration of the reflectance indices in soft sensors, it becomes easier to further involve the ‘speaking-plant’ approach and more physiological parameters, such as Ps, into different control processes. In this approach, deliberately exposing crops to low-dose stress may enhance plant productivity and stress resistance.

Several applications for Machine Learning (ML) do exist, the most significant of which is data mining [14]. Selecting a Machine Learning classifier for a particular task is challenging [15]. Each system is unique, and each system’s effective parameters are likewise unique in terms of magnitude and efficacy. Additionally, many other factors may affect the resulting classification performance, including the characteristics of the training data, user-defined parameter settings, and the predictor variable feature space. According to the feature characteristics, for instance, if continuous, categorical/discrete or binary data are processed, a different algorithm is preferred.

Maxwel et al. [16] sustained that among a series of applicable ML algorithms, the most popular mature ML algorithms are the Support Vector Machine (SVM), the single Decision Tree (DT), the Random Forest (RF), the Boosted Decision Tree (DT), the Artificial Neural Network (ANN), and the *k*-Nearest Neighbor (*k*-NN). According to Maxwel et al. [16], SVM, RF, and Boosted DT have been shown to be very powerful methods for classification of remotely sensed data, and in general, these methods appear to produce overall accuracies that are high compared to alternative machine classifiers such as single DT and *k*-NN. Among them, Boosted DT was found to be more robust and attractive in small size of datasets, producing high classification accuracy.

Generally, the sample size and quality of training data have an important impact on classification accuracy. As a result, it is preferable to obtain a large number of high-quality training samples which fully characterize the class signatures. In greenhouses, however, there are practical limitations to collecting large and error-free training samples. Usually, in those systems, the training sample is small in number and data quality is uncertain due to climatic effect in the remote-sensing process. In this regard, an algorithm that is robust to these issues should be used, such as Boosting DT methods. ANN algorithms also perform well under greenhouse conditions, as they can be applied to complex non-linear problems [17]. Usually, ANN works well with large input data, but it also provides the same accuracy in a smaller dataset.

The aim of this research was to improve the greenhouse control system adjusting both climate and crop physiological data. In this sense, robust ML models were developed (Multilayer Perceptron (MLP) and Gradient Boosting (GB)) based on Artificial Neural Network and the Classification Tree technique. The innovative part of the model is that it uses Ps data measured remotely, using a photochemical reflectance index (PRI), based on a soft sensor. Here, the measured Ps data were combined with climate and leaf temperature data using ML models, something that, to the best of our knowledge, is done for the first time. The specific algorithms were chosen because they can be built quickly to run models that could be easily connected to existing operational control systems.

## 2. Materials and Methods

### 2.1. Greenhouse Crop and Facilities

The experiments were carried out from May to December of 2020 and from May to September of 2021, in one of the six compartments of a gothic type greenhouse. The total ground area of the greenhouse was 1500 m^2^ (250 m^2^ for each compartment). The establishments are located at the facilities of the University of Thessaly, Velestino, Volos (Latitude 39° 22′, longitude 22° 44′ and altitude 85 m), in the continental area of eastern Greece. The greenhouse is covered by a highly transparent polyethylene film and is equipped with a pad and fan system, a thermal screen, and heating system. Air temperature and relative humidity were automatically controlled using a climate control computer (SERCOM, Automation SL, Netherlands) to achieve optimal indoor climate conditions.

The tomato plants (*Solanum lycopersicum* cv. Elpida) were cultivated in slabs filled with perlite (Perloflor Hydro 1, Nordia S.A.; Avlida, Greece). Six crop lines per compartment of 20 m in length and 25 cm in width were cultivated (19 slabs per line, three plants per slab).

The plants were fertigated with a nutrient solution with set points of Electrical Conductivity (EC) around 2 dS m^−1^ and pH of 5.8. The nutrient solution supplied to the crop was a standard nutrient solution for tomato grown in open hydroponic systems adapted to Mediterranean climatic conditions, with the following composition: 5.2 mM L^−1^ Ca^2+^, 2.9 mM L^−1^ Mg^2+^, 2.5 mM L^−1^ K^+^, 1.5 mM L^−1^Na^+^, 11mML^−1^NO_3_^−^, 0.8 mML^−1^ H_2_PO_4_^−^, 23.50 µmM L L^−1^ Fe, 5.00 µmML^−1^ Mn, 3.80 µmML^−1^ Zn. Moreover, the following micronutrients were added to the nutrient solution: chelated with Ethylenediamine tetraacetic acid (EDTA) containing Fe 6%, Mn 13%, Zn 15%, B 21%, Cu 0.3%, Mo 0.2%. The nutrient solution was supplied via a drip system and was controlled by a time-program irrigation controller (8 irrigation events per day at 07:00, 10:00, 12:00, 14:00, 16:00, 18:00, 19:30 and 03:30, local time; dose 120 mL per plant).

### 2.2. Experimental Set Up

To build the ML model, recordings of the plants’ physiological responses to their surrounding microclimate were collected. The tomato plants were exposed to extreme cultivation conditions, such as (i) low air temperature (LTS treatment), (ii) high air temperature (HTS treatment), (iii) low nutrient concentration (LNS treatment) in the root zone and (iv) low water concentration in the root zone (LWS treatment). Additionally, measurements in (v) of non-stressed (NoS treatment) plants were also recorded.

During the LTS recordings, the air temperature was below 18 °C, while the mean value was around 15 °C. The air temperature during the HTS recordings was above 30 °C (±2). In order to impose heat stress conditions on the tomato crop, the greenhouse pad and fan system was not used during some summer hot days and only roof ventilation was performed to cool the greenhouse. In the rest of the treatments, where the air temperature was not a treatment factor, the air temperature was around 25 °C. The well-fertigated, non-stressed plants, NoS, were irrigated with nutrient solution covering 100% of their water and nutrient requirements. In the LNS treatment, the plants were irrigated with low nutrient concentration: 0.83 mM L^−1^ Ca^2+^, 0.91 mM L^−1^ Mg^2+^, 2.5 mM L^−1^ K^+^, 1.5 mM L^−1^ Na^+^, 2.48 mM L^−1^ NO_3_^−^, 0.17 µmML^−1^ Fe. Furthermore, in the LWS treatment, the irrigation was reduced by 75% compared to that of the non-stressed plants. In this regard, the irrigation dose decreased from 120 mL per plant to 30 mL per plant. In parallel, the concentration of the nutrients in the irrigation solution increased accordingly to reach the target concentration.

The tomato transplanting was performed at the stage of two fully expanded leaves, while climate and crop physiological measurements started when the crop leaf area index was approximately 4. To avoid duplication of different types of stress in the cultivation, each treatment was conducted in a different compartment of the greenhouse. The measurements started 10 days after treatment initiation and lasted for 20 days. During the experimental period, measurements of air temperature (T_a_; °C), relative humidity (RH; %), solar radiation (SR; W m^−2^), leaf temperature (T_L_; °C) and photochemical reflectance index (PRI) were collected. The crop measurements were carried out in young and fully developed leaves between the 3rd and 6th branch of three adjacent tomato plants. A total of 13,828 data samples of known type of stress were collected over the period of measurements.

### 2.3. Data Collection and Pre-Processing

**Figure 1:**
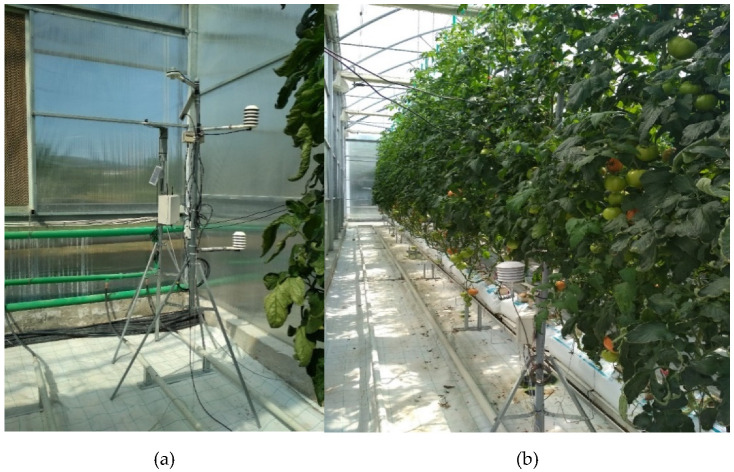
(**a**) The set-up of the multi-sensory tower, (**b**) the tower established within the greenhouse.

A multi-sensor tower was built in order to record crop physiological and microclimate data (Figure 1b). The tower consisted of two air temperature (Thygro SDI-12, Symmetron, Gerakas, Greece) and relative humidity (Thygro SDI-12, Symmetron) sensors, a solar radiation (SP-SS, Apogee Instruments, Logan, UT, USA) sensor, leaf temperature sensors (Thermocouples, type T, Delta Ltd., Eastvale, CA, USA) and a PRI sensor (type SRS-PRI, Meter group, Pullman, WA, USA) (Figure 1a).

The air temperature and relative humidity sensor was placed 1.20 m above the ground to record the microclimate data in the studied crop zone. The solar radiation was placed 2.0 m above the ground. Four thermocouples were attached to young and fully developed leaves of the treated plants and the mean value was recorded.

The remote PRI sensor consisted of an up-looking (incident radiation) and down-looking (reflected radiation) sensor. The two sub-sensors were radiometrically calibrated by default to a NIST-traceable standard and they were centered at 532 nm and 570 nm with 10 nm FWHM. The corrected PRI was calculated as the ratio between reflected and incident radiation, measured by down-looking and up-looking sensor, respectively. The sensor readings upwards and downwards are PRI outputs (Equation (1)):

PRI = (R_531_ − R_570_)/(R_531_ + R_570_), (1)

where R is the radiation reflectance (W m^−2^ nm^−1^) at the specific wavelength. The up-looking SRS sensor was mounted 2 m above the canopy with an unobstructed view of the sky. The down-looking SRS sensor was placed 1.5 m above the ground at a distance of 0.20 m from the crop. The sensor was in a constant 45° angle from the vertical axis to view the zone of young, fully developed leaves. The surface area sensed was approximately 2000 mm^2^. The calibration procedure of the remote PRI sensor followed is presented in detail by Elvanidi and Katsoulas [13].

According to the calibration process, only the PRI sensor values measured during periods with solar radiation values above the crop area higher than 100 W m^−2^ were considered. That is why only measurements between 6:30 and 18:30, local time, during each 20-day period were considered. Values for which the SR was below 100 W m^−2^ during the reported period (from 6:30 to 18:30, local time) were also removed.

To validate the PRI values recorded remotely (PRI_R_), measurements of the PRI values in contact with the leaf (PRI_L_) were also obtained by a hand-held sensor (PlantPen PRI Meter, Alpha Omega-Electronics, Madrid, Spain). Additionally, to correlate the PRI_R_ values with the photosynthesis rate (P_s_mes_; μmol m^−2^ s^−1^), measurements were taken (P_s_mesP_) using a portable photosynthesis system (LCpro, ADC Bioscientific Ltd.; Broxbourne, UK). All measurements were performed in a sample of 20 leaves, under different climatic and light intensity conditions. The regression equation of the 1st order that occurred between the datasets was used to calculate the daily variation of P_s_mes_ and PRI_L_ for the reported 20-day period.

### 2.4. Calculations

To further validate the accuracy of the P_s_mesR_, the recorded values were correlated with P_s_ calculated (P_s_cal_) according to the following relationship:

P = (αI + Pmax − sqrt((αI + Pmax)^2^ − 4θαIPmax))/2θ, (2)

where P is the calculated photosynthesis (P_s cal_); α is light-limited quantum efficiency (~0.5 µmol (CO_2_) µmol^−1^ (photons)); I is the incident PAR radiation in µmol (photons) m^−2^ s ^−1^ (in the PAR spectrum 1 W m^−2^ ~ 2 µmol m^−2^ s ^−1^); Pmax is the maximum photosynthesis rate (here considered equal to 30 µmol CO_2_ m^−2^ s ^−1^), and θ is the convexity (here considered equal to 0.7, dimensionless) [18]. This model is known as nonrectangular hyperbola model, and is used to fit the light-response curves and to estimate photosynthetic parameters. The Pmax was estimated and calculated by means of the nonlinear least squares method under high light intensity. The entire process is described in Ma et al. [19] and Thornley [20].

In addition, two thermal indices were calculated using available measurements of leaf and air temperature, among other environmental factors.

The crop water stress index CWSI is defined as [21]

CWSI = (T_c_ − T_m_)/(T_M_ − T_m_), (3)

where T_c_ is the canopy temperature (°C); T_M_ (°C) is the higher limit of canopy temperature which was assumed to be achieved with minimum canopy conductance, and T_m_ (°C) is the lower limit of canopy temperature which was assumed to be achieved with maximum canopy conductance, estimated as described in Katsoulas et al. [22].

The stress degree day index SDD is defined as [23]

(4) $$\mathrm{SDD}=\sum_{i=1}^{n}(\mathrm{Tc}-\mathrm{Ta}{)}_{i}$$

where T_c_ and T_a_ are the canopy and air temperature at the given time I for the n days of the growing season. Usually, when the index takes positive values, there is an indication of crop stress.

### 2.5. Machine Learning Model

**Figure 2:**
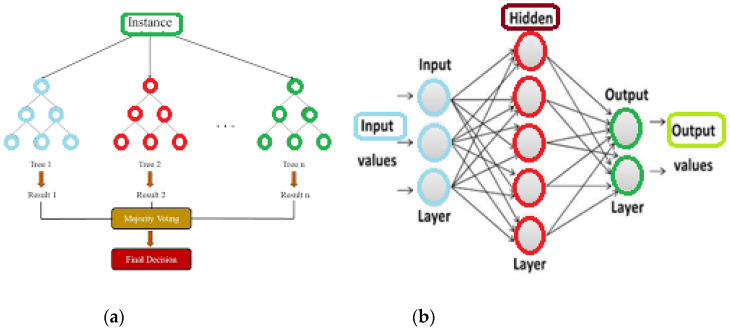
(**a**) Gradient Boosting structure and (**b**) MultiLayer Perceptron structure.

In order to develop a method that predicts different types of crop stress in real time under greenhouse conditions, two Machine Learning (ML) models of two different ML architectures were created. The first model belongs to the category of Neural Networks, specifically the Multilayer Perceptron (MLP) architecture, while the second model belongs to the category of Decision Trees, specifically the Gradient Boosting (GB) (Figure 2).

Both of these models were selected because they were able to treat measured parameters as discrete and not as continuous data such as time-series. In greenhouses, since only the data in which the solar radiation intensity was above 100 W m^−2^ were used, it was necessary to classify the data according to their structure (qualitative or quantitative evaluation). Additionally, these models allow to capture high-order dependencies even when the number of inputs parameters is small. Y. Bengio and S. Bengio [24] studied a series of experiments on modeling the distribution of several discrete datasets and showed that MLP performed statistically significant improvements over other methods such as naive Bayes and comparable Bayesian networks. Similar to MLP, GB has improved the ability to train on categorical variables with high representative learning capacity [25].

Here, it should be mentioned that this was the first time when both MLP and GB models were used under greenhouse conditions for the detection of different types of crop stress, namely NoS(0), HTS(1), LTS(2), LNS(3) and LWS(4).

To build the ML models, two runs were performed. In the first run (RUN 1), 7 qualitative characteristics (SR, Ta, RH, TL, Ps, SDD, CWSI) were used to create the database under the different type of the crop stress. To simplify the above models, a second run (RUN 2) was conducted using 4 qualitative characteristics (Ta, RH, Ps, TL,). Τhe careful selection of inputs, network architecture and learning methods is supported by Karamoutsou [26].

In this case, the scale invariance such as Receiver Operatic Characteristics (ROC) and area under the ROC curve (AUC) was undesirable, since precise calibrated profitability output was required [27]. Probability predictions are based on training data and the distribution of probabilities is compared to the expected probabilities and adjusted to provide a better correspondence. This often involves splitting a training dataset and using one portion to train the model and another portion as a validation set to scale the probabilities.

Here, to set up the models, the total sample of 13,828 datasets was randomly split into 80% training validation (11,062) and tested 20% (2766) sets. To transform, however, probabilities in the training set of known inputs and outputs, weighted least squares regression model was used. Through this process, various values of the weights and biases were checked to determine the set of values so that the computed output values would most closely match the known. Once the training was complete, the accuracy of the resulting model’s weights and biases were applied only once to the test set. As such, the test set was used to select the best performing algorithm and compare its advanced methods without being involved to the learning process. A comparison of the metrics between the training and the test phase indicated that overfitting was avoided. In both models, 10-fold cross validation was examined, since, according to Berrar [28], it usually offers optimum accuracy and better model performance in small and limited dataset.

All steps, learning and classification were written in Python. To support ML algorithms, the Python ML Scikit-learn [29] library and the Spyder environment were used.

#### 2.5.1. Gradient Boosting

GB models are part of the ensemble learning algorithms that rely on a collective decision from inefficient prediction models called decision trees. In the boosting phase, every new tree is a fit on an altered version of the original data set (Figure 2a). Firstly, GB trains a decision tree and assigns each observation an equal weight. Next, after the first tree assessment, the weights for the easy-to-classify observations lower and the weights of the hard-to-classify observations increase. Then, the next tree grows on the weighted data attempting to improve the predictions of the first tree. The new model is now an ensemble of the first and second trees. The classification error is computed, and a third tree is created to predict the revised residuals. This process is repeated for a specified number of iterations until convergence. The prediction of the final ensemble model is the weighted sum of the predictions made by all the previous model iterations.

The methodology and cross validation that used to define iterations is described in Friedman et al. [30], Khan et al. [31] and Ilay Adler and Painsky [32]. For the development of the current GB-based classifier, two main categories of hyperparameters, boosting and tree-specific, were used. Boosting hyperparameters contain learning rate and number of estimator parameters, and tree-specific hyperparameters contain max tree depth and max feature parameters.

The learning rate controls the ease with which the algorithm performs the gradient descent by evaluating the contribution of each tree to the final result. The range of values was 0–1, with the most common values being 0.001–0.3. Smaller values make the model robust to the specific characteristics of each individual tree and reduce the possibility of overfitting. However, they increase the risk of not reaching the optimum with a fixed number of trees and are more computationally demanding. For the development of the current GB-based classifier of both runs, the specific list of learning values [0.05, 0.075, 0.1, 0.25, 0.5, 0.75, 1] was given. The number of estimators comprises the total number of sequential trees to be modeled. Similarly, a list of the number of estimator values was given to the current GB-based classifier of both runs, specifically [10, 20, 30, 40, 50, 60, 70, 80].

The max tree depth controls the depth of the individual trees. In the current algorithm of RUN 1, the specific list of max tree depth values [1, 2, 3, 4, 5, 6, 7, 8] was used in order to find the best max tree depth for the classifier. In RUN 2, the list depth value increased up to 10. Additionally, the max features parameter (which is the number of features that will be used for a best split) was defined in RUN 1 by a specific list of max feature values: [1, 2, 3, 4, 5, 6, 7, 8]. In RUN 2, the values reached up to 4.

The resulted GB classifier was the optimum combination of all the list values for each hyperparameter. In RUN 1, the optimum combination was equal to 0.5 for learning rate, 70 for number of estimators, 9 for max tree depth and 3 for max features. In RUN 2, the best combination of hyperparameters which improve the classifier to classify the five types of stress based on the 4 features values was the following: learning rate 0.5, number of estimators 70, max tree depth 8 and max features 1.

#### 2.5.2. Multilayer Perceptron

MLP is a class of feed-forward Artificial Neural Networks (ANN). The term MLP is used to refer to networks consisting of multiple layers of perceptron (with threshold activation) (Figure 2b). MLP consists of at least three layers: an input layer, a hidden layer, and an output layer. In addition to the input nodes, each node is a neuron that uses a nonlinear activation function. MLP uses a supervised learning technique called backpropagation for training. Its multiple layers and nonlinear activation distinguish MLP from a linear perceptron. Thus, it can distinguish data that is not linearly separable, as required in this study.

In the current study, to develop the MLP-based classifier, two main categories of hyperparameters, hidden levels and a maximum number of iteration lists, are used. The methodology and cross-validation used to define iterations is described in Pal and Mitra [33], Wang et al. [34] and Taki et al. [3].

Hidden layer sizes represent the number of neurons used in each hidden layer. To develop the MLP-based classifier in both runs, three levels were combined, and each of them was assigned the specific list of hidden layer sizes: [1, 2, 3, 4, 5, 6, 7, 8, 9, 10, 20, 30, 40, 50, 60, 70, 80, 90, 100].

The solver iterates until convergence or this number of iterations. Concerning the optimizers, (a) the Stochastic Gradient Descent (SGD) and (b) Adaptive moment estimation (Adam) are tested. For stochastic solvers (‘sgd’, ‘adam’), note that this determines the number of epochs (the number of times each data point will be used), not the number of gradient steps. Therefore, a list of maximum number of iteration values to MLP-based classifier of both runs was assigned, specifically [1, 2, 3, 4, 5, 6, 7, 8, 9, 10, 20, 30, 40, 50, 60, 70, 80, 90, 100, 200, 300, 400, 500, 600, 700, 800, 900, 1000].

The ranges were chosen to test the appropriate variable transformation performed on the sensitive range of the sigmoid transfer function in order to avoid overfitting. If the data used with MLP are not scaled to an appropriate range, the network will not converge on training or it will not produce meaningful results. In this sense, to create the MLP classifier, all the list values for each hyperparameter were combined, and the best combination was chosen. As a result, in RUN 1, the best combination of hyperparameters which improve the current classifier to classify the different types of stress based on the 7 features values was the following: hidden layer sizes (70, 70, 70) and maximum number of iterations 200. In RUN 2, the best combination of hyperparameters which improve our classifier to classify the five types of stress based on the 4 features values is the following: hidden layer list 40 and maximum number of iterations 200.

### 2.6. Performance Evaluation Criteria

Comparison of means of each factor between the treatments was performed by applying one-way ANOVA at a confidence level of 95% (*p* ≤ 0.05) and t-test using SPSS (Statistical Package for the Social Sciences, IBM, USA). The mean values along with the standard deviation (±SD) of the parameters measured are reported.

Usually, in the models that simulate numeric parameters, the Mean Absolute Percentage Error (MAPE), the Mean Absolute Error (MAE), the Mean Squared Error (MSE) and R squared score (R^2^) are analyzed. In the current study, however, which treat both quantitative and qualitative data, the statistical criteria concern only the Accuracy, Positive predictive values (PPV or Precision), Sensitivity (or Recall) and F1 (F1-score) (where P is the number of real positive cases in data and N the number of real negative cases in data):

Accuracy = TP + TN/(P + N),(5)

Precision = TP/(TP + FP),(6)

Sensitivity = TP/(TP + FN),(7)

F1 = 2 (Precision * Sensitivity)/(Precision + Sensitivity).(8)

## 3. Results

### 3.1. Evaluation of PRI and P_s_ Data

**Figure 3:**
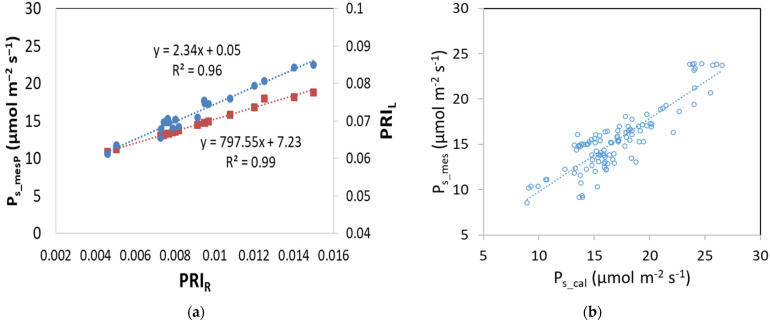
Relationship between (**a**) PRI_R_ with P_s___mes_ and PRI_L_ (square: P_s_ vs. PRI_R_; dot: PRI_L_ vs. PRI_R_) and between (**b**) P_s_mes_ and P_s_cal_.

By plotting PRI_L_ (measured with hand held sensor) and P_S_mesP_ (measured with the portable photosynthesis system) with PRI_R_ values (measure remotely), a linear relationship was found (Figure 3a). Additionally, a linear relationship was also found between P_S_mes_ (calculated with PRI_R_ values) and P_S_cal_ (calculated with the model) (Figure 3b).

**Figure 4:**
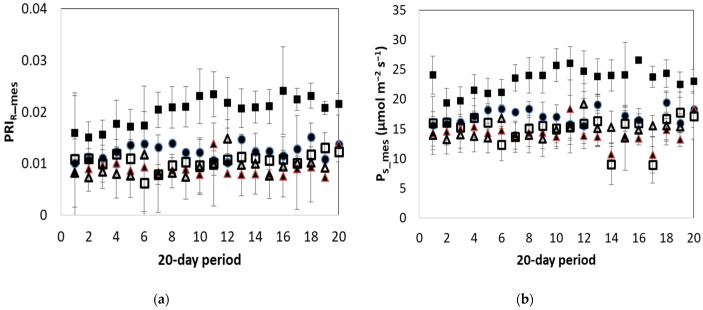
Daily mean values (and their standard deviation) of measured (**a**) photochemical reflectance index (PRI_R_mes_) and (**b**) net photosynthesis rate (P_s_mes_; μmol m^−2^ s^−1^) observed for the different treatments. Solid circle: NoS; Empty triangle: LTS; Solid triangle: HTS; Solid square: LNS; Empty square: LWS.

Figure 4a shows the mean daily variation of PRI_R_ during the reported period. The PRI_R_ values of the unstressed plants varied between 0.010 and 0.015. Values of stressed plants were significantly lower (*p* < 0.05) than those of unstressed plants, except for the LNS treatment where the index ranged from 0.015 to 0.025. In the LWS treatment, the mean value of the index was 9% lower than the mean value of the unstressed measurements. In the HTS and LTS treatments, the values of the index were below 25% of the unstressed plants (*p* < 0.05).

Figure 4b shows the mean daily variation of P_s_mes_ during the reported period. The mean daily value of the non-stressed plants was approximately 17 μmol m^−2^ s^−1^. The relevant values for HTS, LTS and LWS treatments were 16%, 19% and 8% lower than the values observed in the non-stressed plants (*p* < 0.05). In the LNS treatment, the values were 40% higher than those of the non-stressed plants.

**Figure 5:**
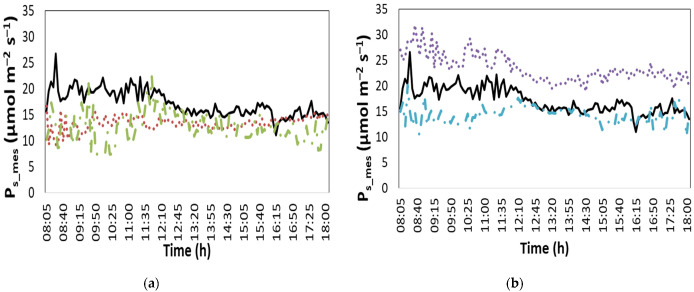
Daily time series of the measured photosynthesis rate on the 10th day of the 20-day reported period (P_s_mes_; μmol m^−2^ s^−1^) (**a**) in NoS, HTS and LTS treatment and (**b**) in NoS, LNS and LWS treatment. Solid line: NoS, dot line: (**a**) LTS; (**b**) LNS, dash-dot: (**a**) HTS; (**b**) LWS.

Figure 5 presents a time series variation of the photosynthesis rate (P_s_, μmol m^−2^ s^−1^) measured online. According to the data, the maximum difference between the stressed treatments and the unstressed plants occurred between the morning and noon period, with the difference showing a decreasing tendency through the day.

### 3.2. Evaluation of Leaf Temperature Data

**Figure 6:**
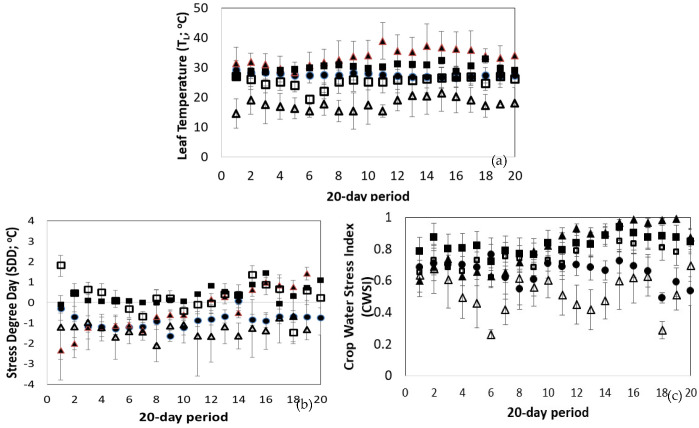
The mean daily values and their standard deviation of (**a**) Leaf Temperature (T_L_, °C), (**b**) Stress Degree Day (SDD; °C) and (**c**) Crop Water Stress Index (CWSI) according to the treatment during the 20-day period. Solid circle: NoS; Empty triangle: LTS; Solid triangle: HTS; Solid square: LNS; Empty square: LWS.

Figure 6a shows the mean daily variation of T_L_ for the different treatments. As expected, the higher and lower T_L_ values were observed in the HTS and LTS treatments with the mean values ranging from 33.4 °C to 17.7 °C, respectively (*p* < 0.05). These mean values were 21% higher and 35% lower than the mean temperature values observed in the non-stressed plants (*p* < 0.05). The LWS and LNS treatment showed a temperature 7.5% lower and 5.3% higher than that of the unstressed plants.

However, to assess whether the leaf temperature indicates some type of stress, the T_L_ must be compared with the air temperature of the surrounding crop area. Figure 6b presents the SDD index which is the difference between T_L_ and T_a_. According to the results, it was found that the values of the index in the unstressed plants ranged from −1.6 °C to 0 °C. Similar to the unstressed plants, the index received negative values in the LTS treatment. However, the leaf temperature was much lower than the temperature of the surrounding air, creating a difference of approximately −2.1 °C. In the case of the HTS treatment, the index had negative values following the path of the unstressed plants during the first 10 days of measurements; then, the index took positive values as the heat stress was on progress, reaching up to 1.4 °C. In the cases of LNS and LWS treatments, the index showed stress from the first days of the measurements.

Figure 6c shows the variation of the CWSI indicator according to the treatment. It was found that the index values for unstressed plants ranged from 0.5 to close to 0.6. In the LTS treatment, the CWSI was lower than that of the unstressed plants. This expected reduction was through the low T_a_ observed over the period reported, and not due to the effect of the treatment on canopy physiology. Similarly, the high air temperature values explain the high CWSI values observed during the HTS treatment. For the rest of the treatments (LNS and LWS), the CWSI values were significantly increased compared to those of the NoS treatment (*p* < 0.05). It was noticed that the reported increase was due to the effect of the treatments on crop physiology.

### 3.3. Automation of Crop Stress Detection

**Figure 7:**
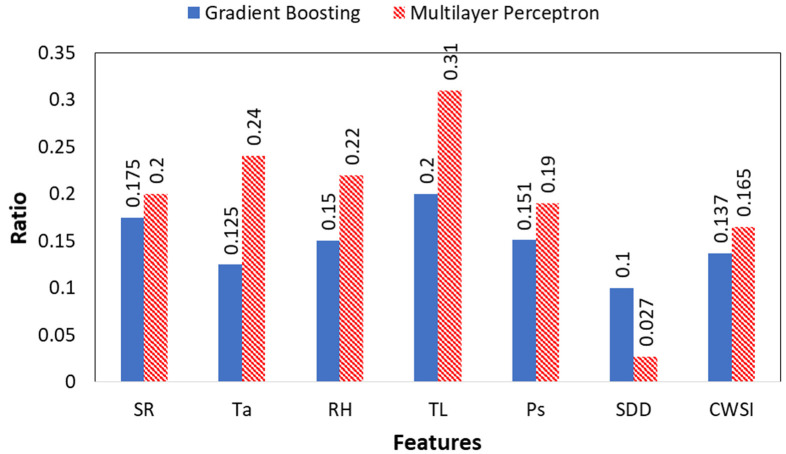
Feature importance of the measured factors in the set-up of GB and MLP algorithm during RUN 1.

Figure 7 shows in the histograms the value importance of the feature obtained from the two ML-based approaches. It is observed that of the 7 features, two features improve the models to classify the five types of stress, T_L_ and T_a_, followed by the features RH, SR and P_s_.

**Figure 8:**
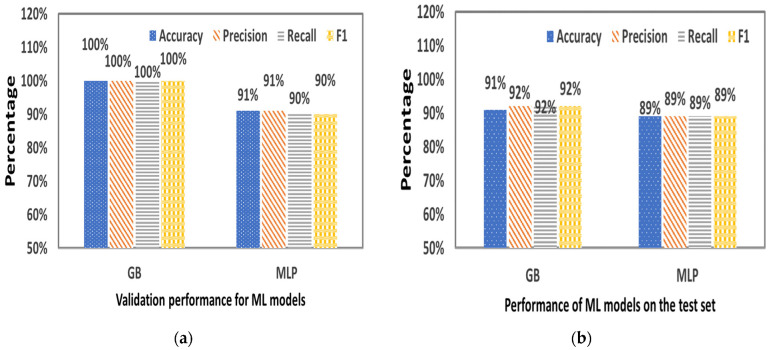
Statistical criteria resulted from (**a**) the validation (training sample 11,062) and (**b**) the performance (testing sample 2765) of GB and MLP algorithm in the RUN 1.

Figure 8 provides the statistical criteria resulting from the validation (Figure 7a) and the performance of the GB and MLP algorithm (Figure 7b) in the RUN 1. Among the two ML algorithms, GB-based classifier was found to be the best performing on the training validation set with 100% Accuracy, 100% Precision, 100% Recall and 100% F1-score. On the other hand, MLP-based classifier performed for the training validation set with 91% Accuracy, 91% Precision, 90% Sensitivity and 90% F1-score. Moreover, GB-based classifier was found to be the best performing on the test set with 91% Accuracy, 92% Precision, 92% Recall and 92% F1-score compared with MLP-based classifier which performed with 89% Accuracy, 89% Precision, 89% Recall and 89% F1-score.

Table 1 shows the performance distribution of the GB model relative to the five types of stress. For example, GB model correctly ‘understood’ 541 cases presented as No Stress, ‘confused’ 46 cases of No Stress with LWS, 20 cases of No Stress with LNS, 4 cases of No Stress with LTS and 1 case of No Stress with HTS. Additionally, according to the data of Table 1, MLP model ‘understood’ 513 cases presented as No Stress, ‘confused’ 61 cases of No Stress with LWS, 28 cases of No Stress with LNS, 12 cases of No Stress with LTS and 12 cases of No Stress with HTS.

### 3.4. Simplification of The ML Models

**Figure 9:**
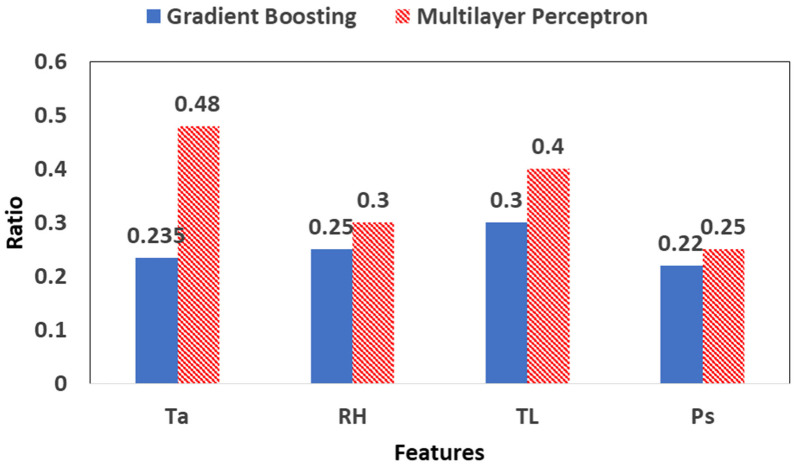
Feature importance of the measured factors in the set-up of GB and MLP algorithm during RUN 2.

The innovative developed models based on GB and MLP algorithm are able to detect all five types of stress with significant results. However, to make the models more robust, it is necessary to decrease the input data by reducing the number of the factors measured in model set-up. The optimum combination of the less measured factors that is able to get involved in the training process is the following: T_a_, T_L_, RH, and P_s_. Figure 9 shows in the histograms the value importance of the feature obtained from the two ML-based approaches in RUN 2.

**Figure 10:**
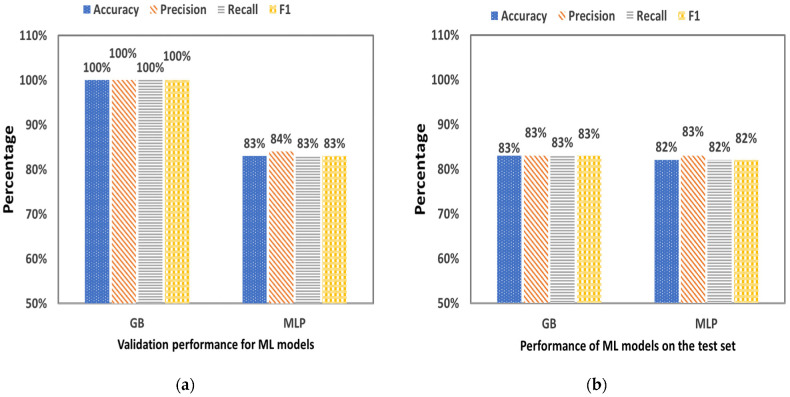
Statistical criteria that resulted from (**a**) the validation (training sample 11,062) and (**b**) the performance (testing sample 2765) of GB and MLP algorithm in the RUN 2.

Figure 10 shows the statistical criteria that resulted from the validation (Figure 9a) and the performance of the GB and MLP algorithm (Figure 9b) in the RUN 2. Among the two ML algorithms, GB-based classifier was found to be the best performing on the training validation set with 100% Accuracy, 100% Precision, 100% Recall and 100% F1-score. On the other hand, MLP-based classifier performed only 83% Accuracy, 84% Precision, 83% Sensitivity and 83% F1-score for the training validation set. Moreover, GB-based classifier was found to have the same performance in the test set compared with MLP, specifically with 83% Accuracy, 83% Precision, 83% Recall and 83% F1-score, while MLP-based classifier performed with 82% Accuracy, 83% Precision, 82% Recall and 82% F1-score.

Table 2 shows the distribution of the performance for the GB model according to the five stress types. According to the data, GB model correctly ‘understood’ 461 cases presented as NoS, ‘confused’ 98 cases of NoS with LWS, 40 cases of NoS with LNS, 7 cases of No Stress with LTS and 6 cases of No Stress with HTS. The less confused predictions were performed in LTS and HTS, while the highest rate of confusion was performed in NoS and LWS treatment. Additionally, MLP model ’understood’ 430 cases presented as No Stress, ‘confused’ 151 cases of No Stress with LWS, 37 cases of No Stress with LNS, 16 cases of No Stress with LTS and 3 cases of No Stress with HTS. Similar to GB model, the less confused predictions were performed in LTS and HTS, while the highest rate of confusion was performed in NoS and LWS treatment.

## 4. Discussion

### 4.1. Assessment of Crop Response

PRI has been used in several studies and has been correlated with rapid changes in de-epoxidation of the xanthophylls cycle and photosynthesis efficiency with very good results [35,36,37]. In the current study, a linear regression equation of 1st order was observed between the remote PRI values and the photosynthesis efficiency values measured with portable gas exchange equipment, in contact with the leaf (Figure 2a). In this way, a large data base of photosynthesis rate on a time scale with high accuracy was developed.

Thus far, only seasonal or diurnal data on changes in photosynthesis have been presented. Bernacchi et al. [38] showed representative measurements of P_s_ on time scale in soybean cultivation under full open-air conditions. They recorded the data, however, by using a conventional portable open gas exchange systems incorporating infrared CO_2_ and water vapor analysers. In this way, they managed to collect measurements from pre-dawn to post-dusk over few days only. Wang et al. [34] also attributed hourly variation in photosynthesis to different types of ecosystems. They estimated the Photosynthetically Active Radiation (PAR) by models using Artificial Neural Network algorithms. Pu et al. [39] and Hu et al. [40] also developed a Neural Network model to predict crop photosynthesis on time scale. Nevertheless, using physiological parameters estimated through ML methods increases the complexity of the final models applied for crop stress detection. Based on the method presented herein, the hourly variation of P_s_ in different climatic and fertigate conditions was obtained. Through this process, measurements were recorded easily, not just within a few days, but for the entire growing season.

According to Bernacchi et al. [38], the photosynthesis rate changes depending on the solar light intensity, so that the maximum values were achieved at noon and the minimum values at pre-dawn and post-dusk time periods. Mohotti and Lwalor [41] also found the same variation in the photosynthesis rate of tea crop. In the current study, in which the plants were cultivated in greenhouse conditions, the P_s_ values were quite higher during morning up to noon. The operation of the cooling and ventilation system used in greenhouses mainly after noon may lead to an increase in the transpiration rate that may lead to initiation of stress conditions contributing to relatively lower values of P_s_. The effect of the greenhouse ventilation system in photosynthesis is verified by Kimura et al. [42].

Photosynthesis values for all the stressed treatments were low, except for LNS treatment. In that treatment, the mechanisms involved in the response and acclimation of photosynthetic CO_2_ assimilation were not activated. Actually, the low nutrients concentration supplied to the plants forced them to develop new strategies for enhancing their growth. Morales et al. [43] asserted that even low concentrations of N in the root zone are able to promote plant growth, further enhancing photosynthesis.

The mechanistic responses to PRI change from the leaf scale to the canopy scale [44,45]. In the current study, a difference between the remote PRI values and the PRI values in contact with the leaf was observed. The difference is explained by the effect of the atmospheric conditions on the remote measurement process. Harris et al. [46] also found the PRI in canopy being weaker from the respective values in leaf-scale.

### 4.2. Evaluation of Developed ML-Based Algorithms

Deep learning technology is maturing day after day. This survey shows that the use of ML in agriculture is huge and has produced remarkable results. There are always challenges, such as creating dataset, time required for training and testing, hardware support, user awareness, etc. However, Internet of Things (IoT) systems and soft sensors combined with ML offer a beneficial solution to improve greenhouse operation. In the current research, real-time parameters of the greenhouse were gathered using climate and crop physiology sensors. The collected data were used by a ML algorithms such as MLP and GB to predict different types of stress.

MLP is a traditional deep learning model that has already been used by researchers in many domains to solve complex problems [47]. In agriculture, it was employed in crop selection, land preparation, seed sowing, irrigation and fertilizing, crop pesticide and harvesting or post-harvesting activities. However, few studies have been conducted in greenhouses and none of them use crop physiological data. Taki et al. [3] used MLP algorithm in greenhouse cultivation system to simulate three different variables such as air, plant and soil temperature. They used as inputs three factors such as the outside air temperature, the wind speed and the solar radiation. The model performed well with the RMSE and MAPE factors ranging from 0.07 °C to 0.12 °C for air temperature and from 0.28 to 0.50%, respectively. Their model helped to further minimize energy consumption. Moon et al. [48] and González-Pérez [49] also used MLP algorithm for indoor greenhouse climate prediction, while Grabarczyk [50] used MLP for modelling heat consumption. Particularly, Moon et al. [48] used in a sample of 87,408 a dataset of eight selected features to predict eight different environmental factors. The RMSE and R^2^ of their air temperature model ranged from approximately 0.83 °C and from 0.97 to 0.99, respectively. González-Pérez and Calderón [49] collected a two-month sample of data (daily data sample of 1440) to develop the MLP model. In their algorithm, eleven inputs were used to predict indoor air temperature and humidity. The model MSE was 1.12 °C for temperature and 6.57% for relative humidity. Grabarczyk [50] added twelve inputs to set up heat consumption modelling. Generally, according to the literature [51], the number of the inputs should be higher or equal to the outputs in order to achieve meaningful results.

MLP is usually chosen to be adopted into precision agriculture workflows, since it provides simple and user-friendly templates for farmers [52]. This is why MLP was selected from the aforementioned researches; however, it requires a large database to work with high accuracy. This is why the MLP algorithm was unable to involve crop data cultivated in greenhouses. In these non-linear systems where there is a lack of datasets and a highly complex dynamic relationship between the climatic factors and the crop physiological response, MLP algorithm is difficult to implement. Recent advances in remote sensing technologies, however, offer a means to overcome some of the limitations of traditional measuring protocols [53].

In the current research, this was the first time that crop data were included in an MLP algorithm. Seven features were selected as input to define five types of stress as output. The resulting accuracy and precision of the training MLP algorithm were sustainable in 91% of the time. This level indicates that the model provided remarkable results in predicting crop stress under the specific dataset sample. Additionally, the comparisons between the predicted and measured definition of crop stress proved satisfactory.

GB algorithm also was used for the first time to rank qualitative and quantitative data to predict the type of crop stress under greenhouse conditions. The model performed higher accuracy and precision from the values of the MLP algorithm. Particularly, the accuracy, precision, sensitivity (or recall) and F1 score in validation performance was 100% instead of 91%, 92%, 91% and 91%, respectively, in the MLP algorithm. That is because the specific algorithm works well under small weak size of datasets and unbalanced data such as real time data management [54,55].

Shyamala and Rajeshwar [51] enhanced the GB regression tree algorithm for crop yield prediction enabling two datasets of size 2000 and 4000 with good accuracy metrics. Khan et al. [31] predicted crop yield using the GB algorithm with a RMSE value of 0.755, MAE of 0.54 and R^2^ value of 0.51. Bhat et al. [55] developed an optimal and highly accurate GB model (based on 300 experimental data points) for rapid and reliable classification of the rose yield environment. In the model, they implemented some of the most influential variables into its architecture, including soil humidity, temperature and humidity of air, CO_2_ concentration, and light intensity (lux).

Additionally, GB construct highly efficient, more accurate, high quality ML models in a lesser amount of time. Ravi and Baranidharan [56] and Cai et al. [57] state that GB is faster compared to all ML algorithms. Cai et al. [57] presented an approach based on GB algorithm to identify a black box model of greenhouse temperature with environment and control data. They noticed that GB can reduce the time required to run the model, with its fast training speed and high accuracy.

However, the process of both models needs to be simplified as farmers prefer to manipulate models that are more autonomous and easier to use. In this sense, an effort was made to minimize the input features ensuring at the same time the high accuracy of the model prediction.

By carrying out all possible combinations that can be achieved in the current research, the 7 features succeeded in being reduced to 4. In the run with less inputs, GB performed higher statistical criteria than the MLP algorithm. This confirms, once again, that GB has worked well under small weak size of datasets, or where there is a call to develop models with fewer inputs than outputs, as in this study. However, an increase in the structure of GB is necessary to further improve their statistical criteria in the test set. The structures and functions used in Neural Networks generally cannot handle all the processes and natural phenomena performed in greenhouse cultivation, as they simplify physics and ‘degenerate’ into weights and threshold values. One has to understand the natural procedures that occur in order to select the appropriate network structure and to apply the appropriate training algorithm.

All the models developed in the current survey could be connected to existing operating systems used in greenhouse. Additionally, these models can be adapted to other greenhouse systems that grow tomato hydroponically, showing a high extensibility in the real application.

This process replaces human experience, allowing farmers to overcome the complexity of the soilless system and guide them toward adopting more sustainable agronomic strategies and technologies. By monitoring crop stress in proper time, the farmers consider the exact needs of the crop, avoiding the mistakes in the operation of the systems ensuring an optimal level of production.

## 5. Conclusions

The approach outlined here addresses the development of machine learning models using remote sensing-based methods that can define the type of crop stress in a non-destructive manner. For this purpose, two different algorithms, Gradient Boosting (GB) and MultiLayer perceptron (MLP), were evaluated based on two runs with different structures. In RUN 1, seven inputs and five outputs were performed to build a model with performance predictive accuracy higher than 91%. To further simplify the model to a more farmer-friendly structure, a run with four inputs and five outputs was performed. In this run, in which the inputs were fewer than the outputs, the performance predictive accuracy decreased to around 82%. To further increase the accuracy level in the second run, the sample size of the dataset should be increased. Both algorithms, however, could be easily connected to existing operating systems used in greenhouses. This improvement will contribute to changing greenhouse control systems, allowing farmers to overcome the complexity of the soilless system. It will guide them toward adopting more sustainable agronomic strategies and technologies, reducing the environmental footprint and saving resources and energy inputs.

## Figures and Tables

**Table 1:** The predicted category of the samples of each treatment for GB and MLP algorithm in the RUN 1 test process (of testing sample 2765).

RUN 1					
	Gradient Boosting				
	No-stress(0)	HTS(1)	LTS(2)	LNS(3)	LWS(4)
No-stress(0)	541	1	4	20	46
HTS(1)	6	520	1	3	33
LTS(2)	0	2	477	0	0
LNS(3)	18	0	1	431	26
LWS(4)	37	19	4	16	560
	**Multilayer Perceptron**				
	No-stress(0)	HTS(1)	LTS(2)	LNS(3)	LWS(4)
No-stress(0)	513	12	12	28	61
HTS(1)	5	504	2	12	39
LTS(2)	1	2	444	0	7
LNS(3)	20	4	0	478	30
LWS(4)	28	17	4	20	523

**Table 2:** The predicted category of the samples of each treatment for GB and MLP algorithm in the RUN 2 test process (of testing sample 2765).

RUN 2					
	Gradient Boosting				
	No-stress(0)	HTS(1)	LTS(2)	LNS(3)	LWS(4)
No-stress(0)	461	6	7	40	98
HTS(1)	14	493	11	5	40
LTS(2)	1	2	464	0	12
LNS(3)	37	4	1	399	35
LWS(4)	95	16	14	35	476
	**Multilayer Perceptron**				
	No-stress(0)	HTS(1)	LTS(2)	LNS(3)	LWS(4)
No-stress(0)	430	3	16	37	151
HTS(1)	14	472	7	7	51
LTS(2)	0	1	449	0	9
LNS(3)	44	5	1	418	47
LWS(4)	34	19	15	31	505

## Data Availability

Data available upon reasonable request.

## References

[1] Toop T.A., Ward S., Oldfield T., Hull M., Kirby M.E., Theodorou M.K. (2017). AgroCycle-Developing a circular economy in agriculture. Energy Proc..

[2] Elvanidi A., Benitez Reascos C.M., Gourzoulidou E., Kunze A., Max J.F.J., Katsoulas N. (2020). Implementation of the circular economy concept in greenhouse hydroponics for ultimate use of water and nutrients. Horticulturae.

[3] Katsoulas N. (2017). EIP-AGRI Focus Group Circular Horticulture: Starting Paper. EIP-AGRI.

[4] Taki Μ., Mehdizadeh S.A., Rohani A., Rahnama M., Rahmati-Joneidabad M. (2018). Applied machine learning in greenhouse simulation; new application and analysis. Inf. Process. Agric..

[5] Escamilla-García A., Soto-Zarazúa G.M., Toledano-Ayala M., Rivas-Araiza E., Gastélum-Barrios A. (2020). Applications of artificial neural networks in greenhouse technology and overview for smart agriculture development. Appl. Sci..

[6] Katsoulas N., Savvas D., Tsirogiannis I., Merkouris O., Kittas C. (2009). Response of an eggplant crop grown under Mediterranean summer conditions to greenhouse fog cooling. Sci. Hortic..

[7] Liakos K.G., Busato P., Moshou D., Pearson S., Bochtis D. (2018). Machine Learning in Agriculture: A Review. Sensors.

[8] Rico-Chávez A.K., Franco J.A., Fernandez-Jaramillo A.A., Contreras-Medina L.M., Guevara-González R.G., Hernandez-Escobedo Q. (2022). Machine Learning for Plant Stress Modeling: A Perspective towards Hormesis Management. Plants.

[9] Ferentinos K.P. (2018). Deep learning models for plant disease detection and diagnosis. Comput. Electron. Agric..

[10] Sengupta S., Lee W.S. (2014). Identification and determination of the number of immature green citrus fruit in a canopy under different ambient light conditions. Biosyst. Eng..

[11] Senthilnath J., Dokania A., Kandukuri M., Ramesh K.N., Anand G., Omkar S.N. (2016). Detection of tomatoes using spectral-spatial methods in remotely sensed RGB images captured by UAV. Biosyst. Eng..

[12] Amatya S., Karkee M., Gongal A., Zhang Q., Whiting M.D. (2015). Detection of cherry tree branches with full foliage in planar architecture for automated sweet-cherry harvesting. Biosyst. Eng..

[13] Elvanidi A., Katsoulas N. (2021). Calibration emthodology of a remote PRI sensor for photosynthesis rate assessment in greenhouses. Biol. Life Sci. Forum.

[14] Kotsiantis S.B., Zaharakis I., Pintelas P. (2007). Supervised machine learning: A review of classification techniques. Emerg. Artif. Intell. Appl. Comput. Eng..

[15] Moud A.A. (2002). Recent advances in utility of artificial intelligence towards multiscale colloidal based materials design. Colloid Interface Sci. Commun..

[16] Maxwell A.E., Warner T.A., Fang F. (2018). Implementation of machine-learning classification in remote sensing: An applied review. Int. J. Remote Sens..

[17] Lavine B.K., Blank T.R. (2009). Chemical and biochemical data analysis. Comprehensive Chemometrics.

[18] Baxevanou C., Fidaros D., Katsoulas N., Mekeridis E., Varlamis C., Zachariadis A., Logothetidis S. (2020). Simulation of Radiation and Crop Activity in a Greenhouse Covered with Semitransparent Organic Photovoltaics. Appl. Sci..

[19] Ma X., Liu Q., Zhang Z., Zhang Z., Zhou Z., Jiang Y., Huang X. (2021). Effects of photosynthetic models on the calculation results of photosynthetic response parameters in young *Larix principis-rupprechtii* Mayr. Plantation. PLoS ONE.

[20] Thornley J. (1976). Mathematical Models in Plant Physiology.

[21] Baille A., Kittas V., Katsoulas N. (2001). Influence of whitening on greenhouse microclimate and crop energy. Agric. For. Meteorol..

[22] Katsoulas N., Baille A., Kittas C. (2002). Influence of leaf area index on canopy energy partitioning and greenhouses cooling requirements. Biosyst. Eng..

[23] Jackson R.D., Idso S.B., Reginato R.J., Pinter P.J. (1981). Canopy temperature as a crop water stress indicator. Water Resour. Res..

[24] Bengio Y., Bengio S. (1999). Modeling high-dimensional discrete data with multi-layer neural networks. Adv. Neural Inf. Process. Syst..

[25] Feng F., Chen H., He X., Sun M., Chua T.S., Ding J. (2019). Enhancing stock movement prediction with adversarial training. IJCAI.

[26] Karamoutsou L. (2020). Investigation of the Water Quality Parameters of Lake Kastoria from Time-Series Monitoring Data Using Machine Learning Techniques for Simulation and Prediction. Ph.D. Thesis.

[27] Klawonn F., Höppner F., May S. (2011). An Alternative to ROC and AUC Analysis of Classifiers. Advances in Intelligent Data Analysis X. IDA.

[28] Berrar D. (2018). Cross-Validation. Encycl. Bioinform. Comput. Biol..

[29] Pedregosa F., Varoquaux G., Gramfort A., Michel V., Thirion B. (2011). Scikit-learn: Machine learning in Python. J. Mach. Learn. Res..

[30] Friedman J.H. (2001). Greedy function approximation: A gradient boosting machine. Ann. Stat..

[31] Khan R., Mishra P., Baranidharan B. (2020). Crop Yield Prediction using Gradient Boosting Regression. Int. J. Innov. Technol. Explor. Eng..

[32] Ilay Adler A., Painsky A. (2021). Feature importance in Gradient Boosting Trees with Cross-Validation feature selection. arXiv.

[33] Pal S.K., Mitra S. (1992). Multilayer Perceptron, Fuzzy Sets, and Classification. IEEE Trans. Neural Networks.

[34] Wang L., Hu B., Kisi O., Zounemat-Kermani M., Cong W. (2017). Prediction of diffuse photosynthetically active radiation using different soft computing techniques. Q. J. R. Meteorol. Soc..

[35] Magney T.S., Eitel J.U., Huggins D.R., Vierling L.A. (2016). Proximal NDVI derived phenology improves in season predictions of wheat quantity and quality. Agric. For. Meteorol..

[36] Katsoulas N., Elvanidi A., Ferentinos K.P., Kacira M., Bartzans T., Kittas C. (2016). Crop reflectance monitoring as a tool for water stress detection in greenhouses: A review. Biosyst. Eng..

[37] Elvanidi A., Katsoulas N., Bartzanas T., Ferentinos K.P., Kittas C. (2017). Crop water status assessment in controlled environment using crop reflectance and temperature measurements. Precis. Agric..

[38] Bernacchi C., Leakey A., Heady L., Morgan P., Dohleman F., McGrath J., Gillespie K., Wittig V., Rogers A., Long S. (2006). Hourly and seasonal variation in photosynthesis and stomatal conductance of soybean grown at future CO_2_ and ozone concentrations for 3 years under full open-air field conditions. Plant Cell Environ..

[39] Pu L., Li Y., Gao P., Zhang H., Hu J. (2022). A photosynthetic rate prediction model using improved RBF neural network. Sci Rep.

[40] Hu J., Xin P.P., Zhang S.W., Zhang H.H., He D.J. (2019). A model for tomato photosynthetic rate based on neural network with genetic algorithm. Int. J. Agric. Biol. Eng..

[41] Mohotti A.J., Lawlor D.W. (2002). Diurnal variation of photosynthesis and photoinhibition in tea: Effects of irradiance and nitrogen supply during growth in the field. J. Exp. Bot..

[42] Kimura K.K., Yasutake D., Koikawa K., Kitano M. (2020). Spatiotemporal variability of leaf photosynthesis and its linkage with microclimates across an environment-controlled greenhouse. Biosyst. Eng..

[43] Morales F., Ancín M., Fakhet D., González-Torralba J., Gámez A.L., Seminario A., Soba D., Ben Mariem S., Garriga M., Aranjuelo I. (2020). Photosynthetic metabolism under stressful growth conditions as a bases for crop breeding and yield improvement. Plants.

[44] Inoue S., Kinoshita T., Matsumoto M., Nakayama K.I., Doi M., Shimazaki K. (2008). Blue light-induced autophosphorylation of phototropin is a primary step for signaling. Proc. Natl. Acad. Sci..

[45] Garbulsky M.F., Penuelas J., Gamon J.A., Inoue Y., Filella I. (2011). The photochemical reflectance index (PRI) and the remote sensing of leaf, canopy and ecosystem radiation use efficiencies: A review and meta-analysis. Rem. Sens. Environ..

[46] Harris A., Gamon J.A., Pastorello G.Z., Wong C.Y.S. (2014). Retrieval of the photochemical reflectance index for assessing xanthophyll cycle activity: A comparison of near-surface optical sensors. Biogeosciences.

[47] Meshram V., Patil K., Meshram V., Hanchate D., Ramkteke S.D. (2021). Machine learning in agriculture domain: A state-of-art survey. Artif. Intell. Life Sci..

[48] Moon T., Hong S., Choi H., Jung D.H., Chang S., Son J.E. (2019). Interpolation of greenhouse environment data using multilayer perceptron. Comput. Electron. Agric..

[49] González-Pérez I., Calderón A. (2018). Neural Networks-based models for greenhouse climate control. J. Automática.

[50] Grabarczyk S. (2018). Modeling of heat consumption in a greenhouse using experimental data. E3S Web Conf..

[51] Shyamala K., Rajeshwar I. (2020). Enhanced gradient boosting regression tree for crop yield prediction. Int. J. Sci. Technol. Res..

[52] Cohen Y., Alchanatis V., Saranga Y., Rosenberg O., Sela E., Bosak A. (2017). Mapping water status based on aerial thermal imagery: Comparison of methodologies for upscaling from a single leaf to commercial fields. Precis. Agric..

[53] Stutsel B., Johansen K., Malbéteau Y.M., McCabe M.F. (2021). Detecting plant stress using thermal and optical imagery from unoccupied aerial vehicle. Front Plant Sci..

[54] Puligudla P., Karthik K.S., Kumar K.V.N., Thirugnanam M. (2020). Prediction of crop yield using gradient boosting. J. Xi'an Univ. Archit. Technol..

[55] Bhat S.A., Huang N.F., Hussain I., Bibi F., Sajjad U., Sultan M., Alsubaie A.S., Mahmoud K.H. (2021). On the Classification of a Greenhouse Environment for a Rose Crop Based on AI-Based Surrogate Models. Sustainability.

[56] Ravi R., Baranidharan B. (2020). Crop yield Prediction using XG Boost algorithm. Int. J. Recent Technol. Eng..

[57] Cai W., Wei R., Xu L., Ding X. (2021). A method for modelling greenhouse temperature using gradient boost decision tree. Inf. Process. Agric..

